# Unsupervised non‐small cell lung cancer tumor segmentation using cycled generative adversarial network with similarity‐based discriminator

**DOI:** 10.1002/acm2.70107

**Published:** 2025-04-23

**Authors:** Chengyijue Fang, Xiaoyang Li, Yidong Yang

**Affiliations:** ^1^ Department of Engineering and Applied Physics University of Science and Technology of China Hefei Anhui China; ^2^ Department of Radiation Oncology the First Affiliated Hospital of USTC Division of Life Sciences and Medicine University of Science and Technology of China Hefei Anhui China

**Keywords:** automated tumor segmentation, cycle‐GAN, lung cancer, unsupervised approach

## Abstract

**Background:**

Tumor segmentation is crucial for lung disease diagnosis and treatment. Most existing deep learning‐based automatic segmentation methods rely on manually annotated data for network training.

**Purpose:**

This study aims to develop an unsupervised tumor segmentation network smic‐GAN by using a similarity‐driven generative adversarial network trained with cycle strategy. The proposed method does not rely on any manual annotations and thus reduce the training data preparation workload.

**Methods:**

A total of 609 CT scans of lung cancer patients are collected, of which 504 are used for training, 35 for validation, and 70 for testing. Smic‐GAN is developed and trained to transform lung CT slices with tumors into synthetic images without tumors. Residual images are obtained by subtracting synthetic images from original CT slices. Thresholding, 3D median filtering, morphological erosion, and dilation operations are implemented to generate binary tumor masks from the residual images. Dice similarity, positive predictive value (PPV), sensitivity (SEN), 95% Hausdorff distance (HD95) and average surface distance (ASD) are used to evaluate the accuracy of tumor contouring.

**Results:**

The smic‐GAN method achieved a performance comparable to two supervised methods UNet and Incre‐MRRN, and outperformed unsupervised cycle‐GAN. The Dice value for smic‐GAN is significantly better than cycle‐GAN (74.5% ± 11.2% vs. 69.1% ± 16.0%, *p* < 0.05). The PPV for smic‐GAN, UNet, and Incre‐MRRN are 83.8% ± 21.5%,75.1% ± 19.7%, and 78.2% ± 16.6% respectively. The HD95 are 10.3 ± 7.7, 14.5 ± 14.6 and 6.2 ± 4.0 mm, respectively. The ASD are 3.7 ± 2.7, 4.8 ± 3.8, and 2.4 ± 1.8 mm, respectively.

**Conclusion:**

The proposed smic‐GAN performs comparably to the existing supervised methods UNet and Incre‐MRRN. It does not rely on any manual annotations and can reduce the workload of training data preparation. It can also provide a good start for manual annotation in the training of supervised networks.

## 1 INTRODUCTION

Lung cancer is the leading cause of cancer‐related deaths according to the World Health Organization.^1^ Computerized tomography (CT) is the most widely used imaging modality for screening, diagnosis and treatment planning of lung tumors.^1^, ^2^ Tumor segmentation which can provide the tumor location and tumor volume is a key step in disease staging and treatment decision‐making,^3^, ^4^, ^5^, ^6^, ^7^ and is also a necessary step in radiotherapy treatment planning.^6^ However, manual segmentation is time‐consuming and relies heavily on the experience of the operators. Automated tumor segmentation methods are therefore in high demand to improve segmentation efficiency.

Among automated tumor segmentation methods, deep learning methods have shown great promise in tumor segmentation due to their ability on learning hierarchical and complex features from medical images. Several networks have been developed to improve performance on lung tumor segmentation^5^, ^8^, ^9^, ^10^, ^11^, ^12^, ^13^. Hossain et al.^13^ introduced a dilation filter into the CNN network, increased the sensing field and segmentation accuracy. Zhao et al.^9^ used an attention module to enhance tumor features and a two‐stage network structure (DRS‐CNN) to tune the delineation results. Jiang et al.^9^ developed a multi‐resolution residual network (Incre‐MRRN)^14^ to improve the generalization capability of the segmentation network to CT images. Yang et al.^14^ proposed a 3D deep supervision‐enhanced U‐Net (MSDS‐UNet^15^) improving tumor segmentation accuracy by capturing inter‐slice continuity, using multi‐level supervision to refine features at different scales. The performance of different supervised methods is listed in Table 1.

**TABLE 1:** Existing supervised methods on lung tumor segmentation.

Methods	Dice	SEN	PPV	$HD_{95}$ (mm)	Train	Val	Test
3D ResUNet	0.667	0.728	0.706	*	269	60	66
MSDS‐UNet	0.691	0.746	0.719	*	269	60	66
FRRN	0.516	0.703	0.489	12.66	269	60	66
Incre‐MRRN	**0.74**	**0.8**	**0.73**	**7.94**	**377**	**304**	**529**
DRS‐CNN	0.644	0.759	0.741	12.44	48	5	20
Dilated‐CNN	0.657	*	*	*	260		40

*Note*: Dice is volume dice. SEN represents sensitivity. PPV represents positive predictive value and $HD_{95}$ represents 95% Hausdorff distance. Train, Val and Test are the numbers of patients in training, validation and test databases. Bold values indicate the highest values in the table.

However, these supervised methods depend on manual annotation for training. It is costly and labor‐intensive to obtain a sufficiently large annotated dataset for training in clinical. To address this limitation, unsupervised tumor segmentation methods^16^, ^17^, ^18^, ^19^, ^20^, ^21^have emerged as a promising alternative, which do not require annotated data. Among these, generative adversarial networks (GANs) offer an advanced approach to tumor segmentation due to their ability on learning complex tumor texture under diverse imaging conditions. Through generating synthetic normal images, GANs can distinguish abnormal regions by comparing synthetic and real images. Schlegl et al.^20^ developed f‐AnoGAN by training generate network only using normal images and identifying deviations as abnormal regions. Voorst et al.^21^ trained a GAN to transform a CT scan with lesions into a generated scan without lesions and extracted lesions from their difference map. Fang^22^ trained a cycle‐GAN to transform CT images with pneumonia lesions of COVID‐19 into synthetic healthy images and segmented lesions by subtracting synthetic healthy CT images from images with lesions.

In this work, we introduce a similarity‐based discriminator into cycle‐GAN (smic‐GAN) to improve the performance of synthetic CT image generation and tumor delineation. Typically, lung tumors exhibit asymmetry and appear in only one lung. While standard discriminators are commonly used in cycle‐GAN training, we propose an additional similarity‐based discriminator that evaluates the generated CT images by comparing the bilateral lungs. After generating synthetic healthy CT images, residual images with tumor information are obtained by subtracting synthetic healthy CT images from real CT images with tumors. The proposed unsupervised method can improve healthy image synthesis and reduce the workload of manual lesion annotating.

## 2 MATERIALS AND METHODS

The proposed tumor segmentation method is performed in three steps. First, smic‐GAN network is trained to transfer CT slices containing tumors (unhealthy) into synthetic CT slices without tumors (healthy). Second, residual images are obtained by subtracting synthetic images from original images. Finally, post‐processing, including threshold segmentation, median filtering and morphological operations, is applied to residual images to obtain binary tumor masks.

### 2.1 Network structure

#### 2.1.1 Smic‐GAN

**FIGURE 1:**
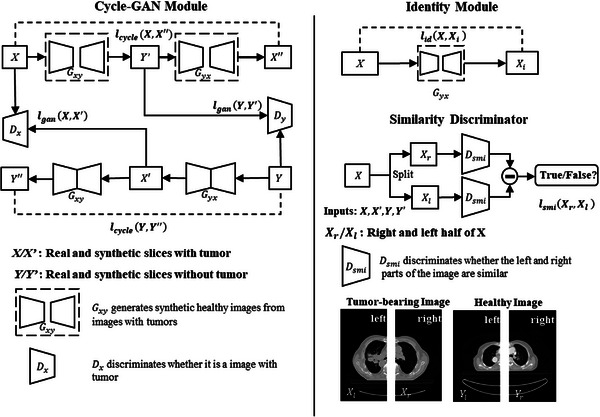
The smic‐GAN structure. $G_{\mathrm{xy}},G_{\mathrm{yx}}$ are generators that transfer between the images with and without tumors. $D_{x},D_{y}$ are discriminators used to distinguish the differences between synthetic images and real images. $D_{\mathrm{sim}}$ is the discriminator to distinguish the left and right lung. $l_{\mathrm{cycle}},l_{\mathrm{gan}},l_{\mathrm{id}}$, and $l_{\mathrm{smi}}$ are four components of the loss function. $l_{\mathrm{cycle}}$ is used to keep consistence of the image. $l_{\mathrm{gan}}$ is the difference between the generated images and real images. $l_{\mathrm{id}}$ is to ensure that only regions corresponding to tumors are changed. The aim of $l_{\mathrm{smi}}$ is to train a discriminator to differentiate healthy and unhealthy images based on the symmetry of bilateral lungs.

As depicted in Figure 1, smic‐GAN consists of three modules: cycle‐GAN module, identity module and similarity discriminator. The cycle‐GAN module consists of two generators $G_{xy}$, $G_{yx}$ and two discriminators $D_{x},\;D_{y}$. $G_{xy}$ generates synthetic healthy CT images from original unhealthy CT images. $G_{yx}$ generates synthetic unhealthy CT images from original healthy images. Two discriminators $D_{x}$ and $D_{y}$ are trained to differentiate the real CT images from the synthetic ones. Secondly, the identity module is designed to prevent unnecessary modifications on original images. Finally, the similarity discriminator $D_{smi}$ ensures that the left and right lungs are similar in either real or synthetic healthy images.

#### 2.1.2 Generator structure

**FIGURE 2:**
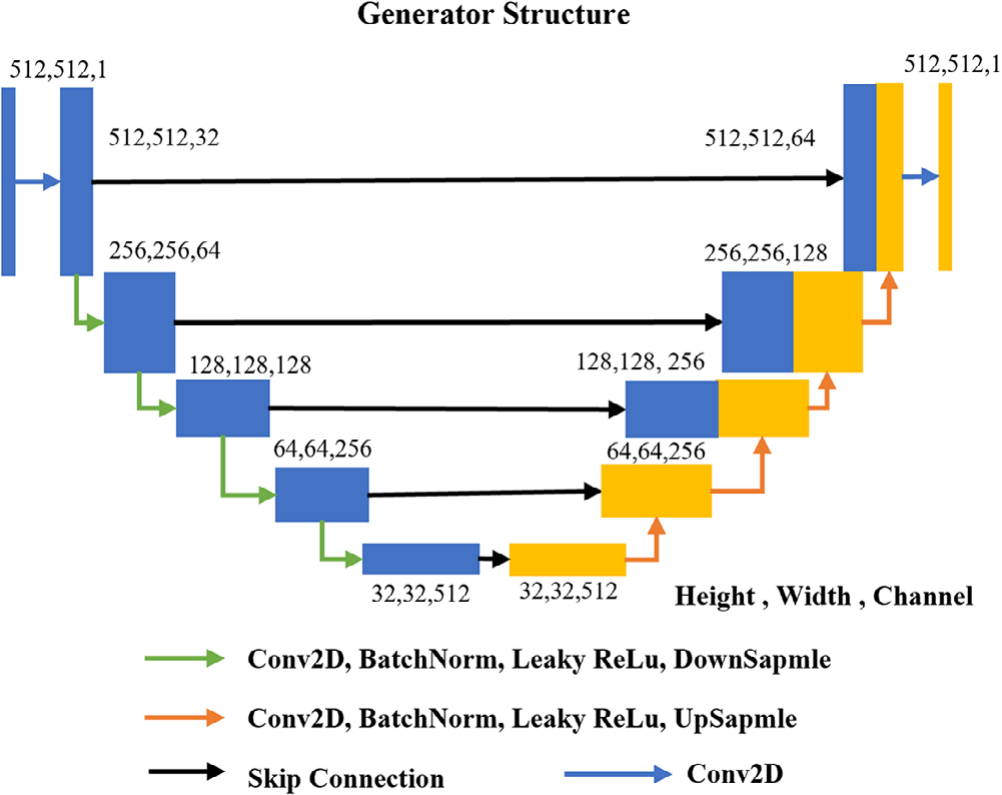
The structure of generators in the smic‐GAN. The input and output of the generator are images having a size of 512 × 512 pixels.

The generator structure ($G_{xy},G_{xy}$) is presented in Figure 2, featuring a symmetric network structure with four down‐ and up‐sampling steps. In the first layer, a feature map with 32 channels is generated from the input image using 2D convolutions. The kernel size of the 2D convolutions is 3 × 3 pixels. The padding step is 1 pixel and the stride is 2 pixels. In the four down‐sampling layers, 2D convolution, batch normalization, leaky‐ReLU activation and down‐sampling are applied. In each down‐sampling step, the height and width are halved and the channel is doubled. In each up‐sampling step, the height and width are doubled and the channel is halved. Skip connections are applied to concatenate each layer in the down‐sampling and up‐sampling process. The output image is generated in output layer using 2D convolutions.

#### 2.1.3 Discriminator structure

**FIGURE 3:**
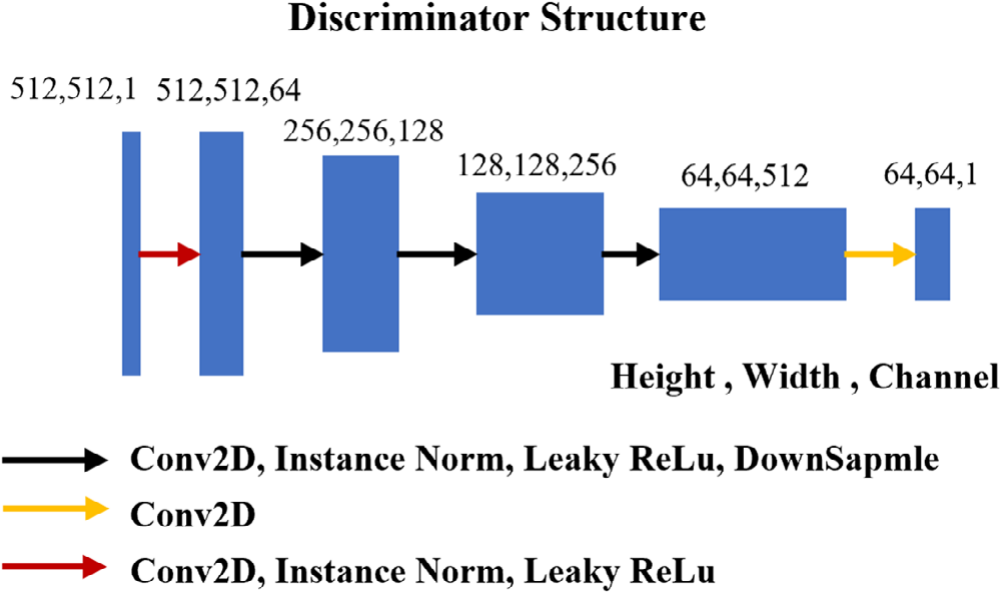
The structure of discriminators in the smic‐GAN. The input is the original image or synthetic image and the output is a 64 × 64 matrix for discriminator loss calculation.

**FIGURE 4:**
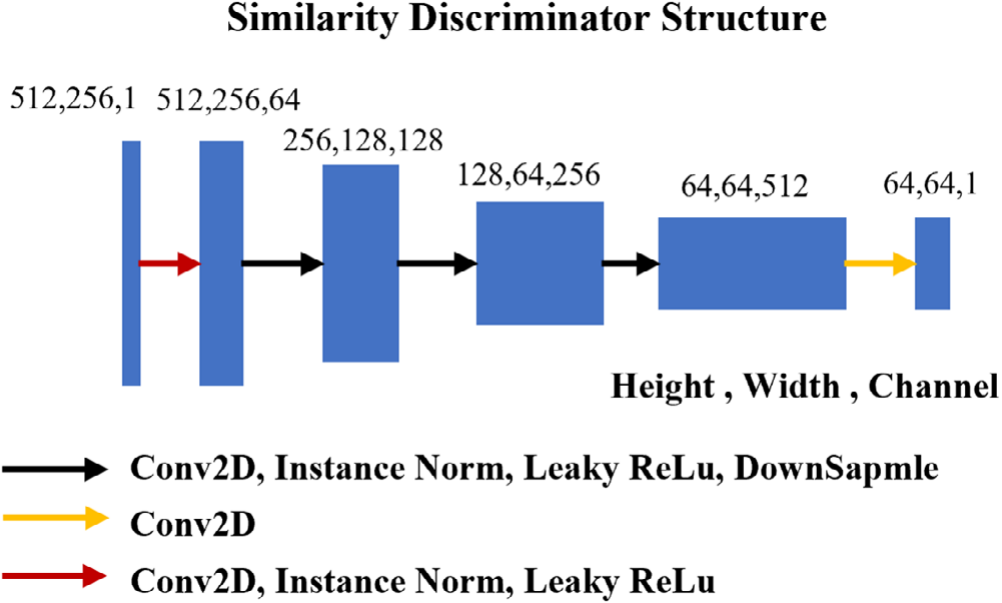
The structure of the similarity discriminator in the smic‐GAN. The input is either the left or right part of the real or synthetic image, and the output is a 64 × 64 matrix for loss calculation.

The structures of the general discriminator ($D_{y}$, $D_{x}$) and similarity discriminator $D_{smi}$ are shown in Figures 3 and 4, respectively. The input image size is 512 × 512 pixels for ($D_{y}$, $D_{x}$) and 512 × 256 pixels for $D_{smi}$. In either discriminator, the first layer expands the input image into a feature map with 64 channels using 2D convolutions. In the second to fourth layers, instance normalization, leaky‐ReLU activation and down‐sampling are applied after the convolution. The output layer uses 2D convolutions to convert the size of feature map into 64 × 64 × 1 for discriminator loss calculation.

### 2.2 Loss function

Three loss functions are used to optimize two generators $G_{xy}$, $G_{yx}$ and three discriminators $D_{x}$, $D_{y}$, and $D_{smi}$, including generator loss $l_{gen}$, discriminator loss $l_{dis}$ and similarity loss $\;l_{smi}$.

The generator loss consists of three parts, including $l_{gan},\;l_{id}$, and $l_{cycle}$, as shown in Equation (1). $\;\lambda_{c}$ and $\lambda_{i}$ are the weights of the cycle loss and identity loss, respectively. The weights are set to ensure that the three loss terms are relatively balanced during training process. In this experiment, $\lambda_{c}$, $\lambda_{i}$ are 10 and 5, respectively.

(1) $$l_{gen}=l_{gan}+\lambda_{c}\cdot l_{cycle}+\lambda_{i}\cdot l_{id}$$

The GAN loss $l_{gan}$ is to minimize the difference between the synthetic and real images, and designed as:

(2) $$\begin{array}{c} l_{gan}=∥\left(D_{y}\left(G_{xy}\left(x\right)\right)-1\right){∥}_{2}+∥\left(D_{x}\left(G_{yx}\left(y\right)\right)-1\right){∥}_{2} \end{array}$$

${\left\|*\right\|}_{2}$ is the $l_{2}$ norm. $G_{xy}\left(x\right),\;G_{yx}\left(y\right)$ are synthetic CT slices. $D_{x}\left(*\right),D_{y}\left(*\right)$ are outputs of the two discriminators. For optimized $G_{xy}$ and $G_{yx}$, the outputs of the discriminators are supposed to be close to 1.

The cycle loss $l_{cylce}$ is used to keep the consistency of the two generators $G_{xy}$ and $G_{yx}$ and defined as:

(3) $$\begin{array}{c} l_{cycle}=∥G_{yx}\left(G_{xy}\left(x\right)\right)-x{∥}_{1}+∥G_{xy}\left(G_{yx}\left(y\right)\right)-y{∥}_{1} \end{array}$$

${\left\|*\right\|}_{1}$ is $l_{1}$ norm. With this constraint, $G_{xy}$ and $G_{yx}$ learn unique mappings between healthy and unhealthy images, avoiding generation collapse.^23^

The identity loss $l_{id}$ is designed to ensure that only the region corresponding to the tumor are changed between healthy and tumor‐bearing images, and designed as:

(4) $$l_{id}=∥G_{xy}\left(y\right)-y{∥}_{1}+∥G_{yx}\left(x\right)-x{∥}_{1}$$

The discriminator loss is defined in Equation (5). The aim is to train a robust discriminator to distinguish real healthy (or unhealthy) images from synthetic healthy (or unhealthy) images. For optimized $D_{x}$ and $D_{y}$, the outputs of two discriminators should be close to 0 when synthetic images are input and close to 1 when real images are input.

(5) $$\begin{array}{cc} l_{dis} & =∥D_{x}\left(G_{yx}\left(y\right)\right){∥}_{2}+∥D_{y}\left(G_{xy}\left(x\right)\right){|}_{2}\\ & \;+∥D_{y}\left(y\right)-1{|}_{2}+∥D_{x}\left(x\right)-1{∥}_{2} \end{array}$$

The similarity loss is defined in Equation (6). The aim of $l_{smi}$ is to train a discriminator to differentiate healthy and unhealthy images based on the symmetry of bilateral lungs.

(6)

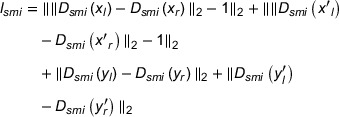

where x, y are real healthy and unhealthy CT images, $x^{\prime }$, $y^{\prime }$ are synthetic ones, respectively. ${*}_{l}$, ${*}_{r}$ are left and right half of the images. For real and synthetic healthy CT slices, the outputs of similarity discriminator $D_{smi}\left({*}_{l}\right)$ and $D_{smi}\left({*}_{r}\right)$ should be close to each other. For real and synthetic unhealthy ones, the absolute value of the difference between $D_{smi}\left({*}_{l}\right)$ and $D_{smi}\left({*}_{r}\right)$ should be close to 1.

The detailed training process is shown in Table 2. The ADAM optimizer with parameters of $\beta_{1}=0.5$ and $\beta_{2}=0.999$ is used. A total of 200 epochs are trained with an initial learning rate of $2e^{-4}.$ The learning rate was kept constant for the first 150 epochs and then linearly decayed to 0 for the remaining 50 cycles. The network is trained using PyTorch on two NVIDIA GTX 4080Ti GPUs.

**TABLE 2:** Algorithm 1: the training process of proposed method.

*# Initialize models and parameters* **Initialize** $G_{xy}$, $G_{yx}$, $D_{x}$, $D_{y}$, $D_{smi}$ Set **hyperparameters**: $\lambda_{c}=10$, $\lambda_{i}=5,$ learning rate $lr=2e^{-4}$, total_epochs = 200, decay_epochs = 50	
1.	**for** epoch **in** range (1, total_epochs) **do**:
2.	**for** each batch of CT slices (X, Y) **do**:
	*# Step 1: Train Generators*
3.	Generate: $Y^{\prime }=G_{xy}\left(X\right)$, X 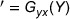
4.	Compute generator loss: $l_{gen}$
5.	Generate: $X^{\prime \prime }=G_{yx}\left(Y^{\prime }\right)$, $Y^{\prime \prime }=G_{yx}\left(X^{\prime }\right)$
6.	Compute cycle loss: $l_{cycle}$
7.	Generate:$Y_{i}=G_{xy}\left(Y\right)$, $X_{i}=G_{yx}\left(X\right)$
8.	Compute identity loss: $l_{id}$
9.	Calculate total loss: $l_{gan}=l_{gen}+\lambda_{c}\cdot l_{cycle}+\lambda_{i}\cdot l_{id}$
10.	Update $G_{xy},G_{yx}$
	*# Step 2: Train Discriminator*
11.	Calculate discriminator loss: $l_{dis}$
12.	Update $D_{x},D_{y}$
	*# Step 3: Train Similarity Discriminator*
13.	Split $X,Y,X^{\prime },Y^{\prime }$ into left and right parts
14.	Calculate similarity discriminator loss: $l_{smi}$
15.	Update $D_{sim}$
16.	e**nd for**
17.	**if** epoch **in** range (total_epochs—decay_epochs, total_epochs) **do**:
18.	Decay learning rate $l_{r}$
19.	**end if**
20.	**end for**

### 2.3 Image postprocessing

After the residual images are obtained by subtracting synthetic healthy CT images from real tumor‐bearing images, the postprocessing is performed in three steps. First, binary tumor masks are segmented using Otsu thresholding. Then, median filtering with a kernel size of 3×3 is applied to denoise the mask. Finally, morphological erosion and dilation operations with a disk of diameter 2 pixels are applied to refine the segmentation results.

### 2.4 Data preparation

The proposed method is trained and evaluated using a combination of two datasets: USTCFAH and NSCLC‐Radiomics.^13^ The USTCFAH dataset includes 187 lung cancer patients who underwent radiation therapy at the First Affiliated Hospital of the University of Science and Technology of China. CT images have a pixel size of 0.977 mm × 0.977 mm and a slice thickness of 1 mm. The scans were acquired with a tube voltage of 120 kVp and a current of 230 mAs. Gross tumor volume (GTV) regions are used as lung tumor masks for analysis. The dataset is divided into training (157 patients), validation (10 patients), and test (20 patients) sets. The public NSCLC‐Radiomics dataset includes 422 patients with non‐small cell lung cancer. CT images have a pixel size of 0.977 mm × 0.977 mm and a slice thickness of 3 mm. The dataset is divided into training (347 patients), validation (25 patients), and test (50 patients) sets. Both datasets are merged into a single unified set, resulting in a combined dataset of 609 patients for training (504 patients), validation (35 patients), and testing (70 patients). The combined training dataset includes 8723 slices with a tumor and 8940 slices without tumors. Among the patients, 345 of them had one tumor in the left lung, and 264 in the right lung.

The CT slices are manually categorized as healthy slices for those without any tumor or unhealthy slices for those bearing a tumor. The pixel intensity of CT images was normalized to the range of [0,1] using the following equation:

(7) $$f\;\left(x\right)=\left\{\begin{array}{c} 1,\;if\;\frac{x}{1500}>1\\ \frac{x}{1500},\;else\;\\ 0,\;if\;\frac{x}{1500}<0 \end{array}\right.$$

where x is the CT number in Hounsfield Unit (HU). This normalization enhances data consistency and mitigates potential variations caused by differences in numerical values.

### 2.5 Evaluation

The results of smic‐GAN are compared with two supervised methods, UNet^13^ and Incre‐MRRN.^9^ UNet is a commonly used supervised method in medical image segmentation. Incre‐MRRN is the state‐of‐the‐art supervised method on lung tumor segmentation of CT images. The smic‐GAN is also compared with cycle‐GAN, a previously published method^22^ for COVID‐19 lesion segmentation. All networks are trained with the same parameter setting and on the same training and validation set.

To evaluate the proposed method, Dice similarity, positive predictive value (PPV) and sensitivity (SEN) are used and defined as follows:

(8) $$Dice\;\left(\%\right)=\frac{2\left(\;V_{pre}\cap V_{gt}\right)}{V_{pre}+V_{gt}}\;\times 100\%$$

(9) $$PPV\;\left(\%\right)=\frac{V_{pre}\cap V_{gt}}{V_{pre}}\;\times 100\%$$

(10) $$SEN\;\left(\%\right)=\frac{V_{pre}\cap V_{gt}}{V_{gt}}\;\times 100\%$$

where $V_{pre}$ and $V_{gt}$ are the predicted and ground truth volume, respectively

The 95% Hausdorff distance ($HD_{95}$) and average surface distance (ASD) are used to evaluate the surface dissimilarity between predicted results and ground truth. They are defined as follows:

(11) $$HD_{95}\;\left(mm\right)=\max_{95\%}\left(\min_{x\in X,y\in Y}d\left(x,y\right),\min_{x\in X,y\in Y}d\left(y,x\right)\right)$$

(12) $$\begin{array}{cc} & ASD\;\left(mm\right)\\ & =\mathrm{mean}\left(max\left(\min_{x\in X,y\in Y}d\left(x,y\right),\min_{x\in X,y\in Y}d\left(y,x\right)\right)\right) \end{array}$$

where *x* and *y* are pixels belonging to the surface of the predicted tumor volume and ground truth, respectively. d(x,y) is the Euclidean distance between *x* and *y*.

## 3 RESULTS

**FIGURE 5:**
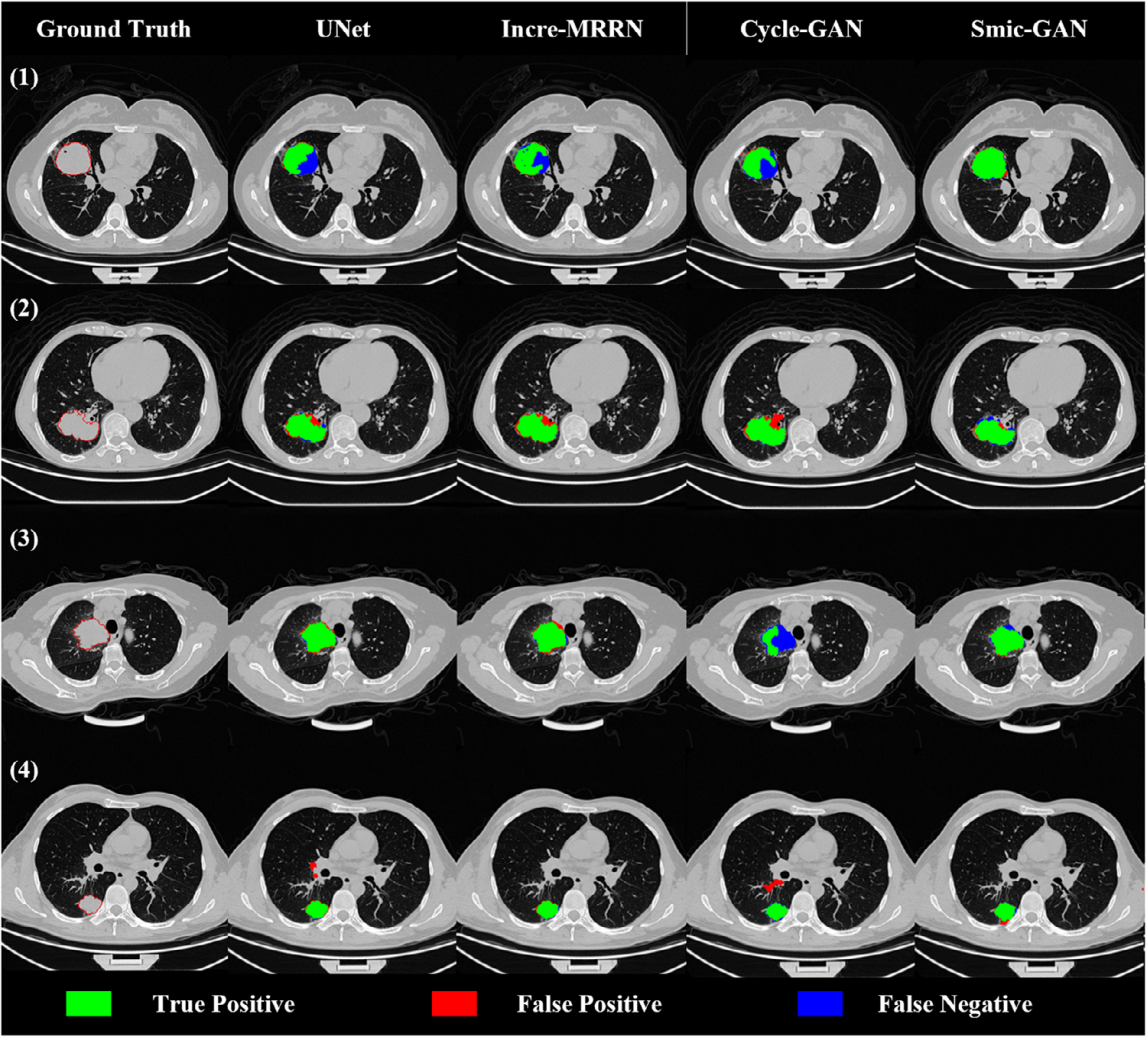
The comparison of segmentation capability of UNet, Incre‐MRRN, cycle‐GAN and smic‐GAN on four patients. The left column is the ground truth. The red, green and blue regions represent for false positive, true positive and false negative volume, respectively.

Tumor delineation results for four patients are shown in Figure 5. For patient 1, the proposed method successfully segments the entire tumor while all three other methods miss some partial tumor volume. For patient 2, the proposed method has least false positive region while all four methods result in similar true positive region. For patient 3, the tumor is close to the trachea, simc‐GAN performs comparably to the supervised methods. The cycle‐GAN has the worst performance in the true positive value. For patient 4, the tumor is small and close to the chest. While all four methods result in similar true positive region, both UNet and cycle‐GAN have larger false positive regions near trachea.

**FIGURE 6:**
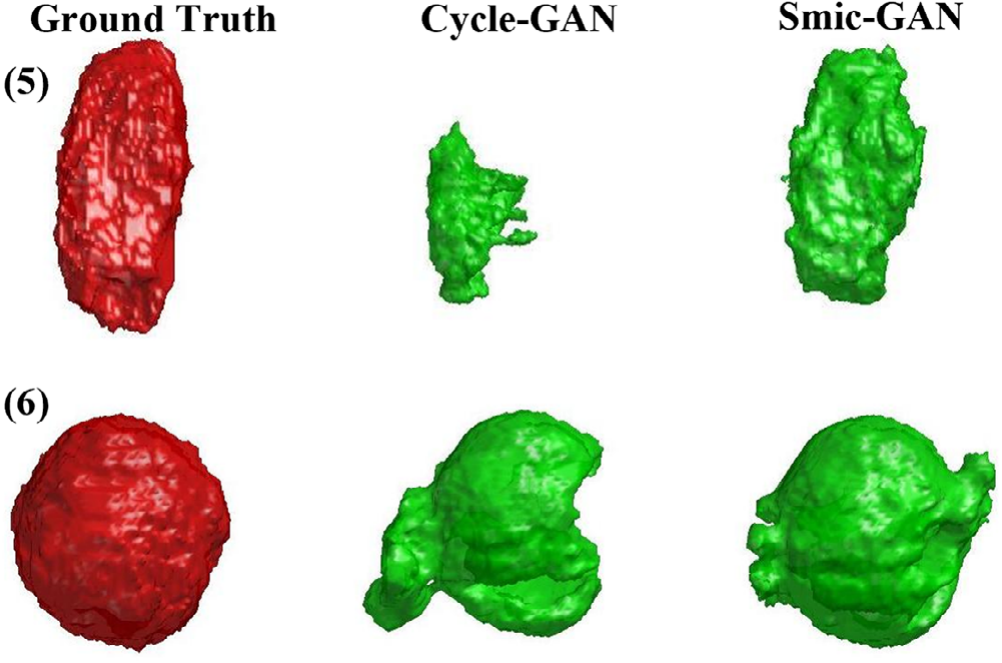
3D surface view of two additional segmentation results using cycle‐GAN and smic‐GAN.

To further compare the two unsupervised methods, cycle‐GAN and smic‐GAN, Figure 6 presents the 3D segmentation results for two additional patients. As shown, smic‐GAN significantly outperforms cycle‐GAN in tumor delineation, achieving much higher Dice scores (81.5% vs. 29.5% for patient 5 and 79.1% vs. 55.0% for patient 6). Notably, cycle‐GAN misses a substantial portion of the tumor in patient 5, resulting in poor segmentation performance. These results demonstrate that smic‐GAN provides more accurate and complete tumor segmentation than cycle‐GAN.

Table 3 presents the Dice, PPV, and SEN values averaged over 70 test patients. The statistical results demonstrate comparable performance between smic‐GAN and the two supervised learning methods on Dice score (74.5% ±11.2% for smicGAN, 77.6% ±11.2% for Incre‐MRRN, 75.8% ±11.6% for UNet). The proposed method outperforms the cycle‐GAN which is currently a state‐of‐the‐art unsupervised method^22^ for region delineation (69.1%±16.0% vs. 74.5% ±11.2%). The paired *t*‐test results between the proposed method and other three methods are presented in Table 4. Table 5 shows the $HD_{95}$ and ASD results and Table 6 the paired *t*‐test results. The smic‐GAN surpasses cycle‐GAN on both $HD_{95}$ and ASD. In addition, the $HD_{95}$ of smic‐GAN (10.3 ± 7.7 mm) is significantly smaller than that of UNet (14.5 ± 14.6 mm), but greater than that of Incre‐MRRN (6.16 ± 4.04 mm). The ASD of smic‐GAN (3.74±2.74 mm) is smaller than that of UNet (4.77± 3.78 mm) but greater than that of Incre‐MRRN (2.42±1.85 mm).

**TABLE 3:** Dice, precision, and sensitivity scores of different methods.

		Supervised		Unsupervised	
Methods		UNet	MRRN	Cycle‐GAN	Smic‐GAN
**DICE (%)**	Mean ± std	75.8 ±11.6	**77.6** ± **11.2**	69.1 ±16.0	74.5 ±11.2
	Median	78.0	**80.1**	7.24	76.5
	IQR	70.2–83.0	**74.3–85.9**	59.8–83.1	70.0–82.5
**PPV (%)**	Mean ± std	75.1 ±19.7	78.2 ±16.6	78.8 ±16.9	**83.8 ± 21.5**
	Median	81.6	81.4	80.4	**92.1**
	IQR	63.4–96.3	70.0–93.0	68.8–90.6	**79.0–97.8**
**SEN (%)**	Mean ± std	77.9 ±17.9	**79.8** ± **17.9**	66.1 ±23.8	63.7 ± 19.0
	Median	81.6	**83.8**	77.3	66.9
	IQR	63.4–96.3	**66.0–96.0**	55.2–87.1	58.1–81.7

*Note*: PPV represents precision. SEN represents sensitivity. IQR is the interquartile range. Std means standard deviation. Bold values indicate the highest values in the table.

**TABLE 4:** *p* value between smic‐GAN and other methods on dice score, precision and sensitivity scores.

Methods	Smic‐GAN vs. UNet	Smic‐GAN vs. MRRN	Smic‐GAN vs. cycle‐GAN
DICE	0.505	0.097	0.023
PPV	0.014	0.087	0.131
SEN	<0.001	<0.001	0.511

**TABLE 5:** $HD_{95}$ and ASD results of different methods.

	Supervised		Unsupervised	
Methods	UNet	MRRN	Cycle‐GAN	Smic‐GAN
$HD_{95}$ (mm)	14.5 ± 14.6	**6.2 ** ± **4.0**	18.1 ± 17.7	10.3 ± 7.7
ASD (mm)	4.8 ± 3.8	**2.4** ± ** 1.9**	6.0 ± 4.7	3.7 ± 2.7

*Note*: $HD_{95}$ represents 95% Hausdorff distance and ASD means average surface distance. Bold values indicate the highest values in the table.

**TABLE 6:** *p* value between proposed method and other methods.

Methods	Smic‐GAN vs. UNet	Smic‐GAN vs. MRRN	Smic‐GAN vs. cycle‐GAN
$HD_{95}$	0.024	<0.001	<0.001
ASD	0.067	<0.001	<0.001

*Note*: $HD_{95}$ represents 95% Hausdorff distance and ASD means average surface distance.

## 4 DISCUSSION

In this work, a GAN‐based network, smic‐GAN, is developed for automatic lung tumor segmentation by leveraging the symmetry constraint based on the inherent symmetricity of human lungs, and enables enhanced tumor delineation accuracy. Although it still requires categorizing CT slices into healthy and tumor slices as separate inputs, smic‐GAN does not require manual data annotation in the network training process and thus reduces the data preparation workload. Simc‐GAN enables clinics to develop customized segmentation models using their own data with minimal annotation efforts and can provide an initial sketch as a good start for manual annotation in the training of supervised networks like UNet and Incre‐MRRN.

**FIGURE 7:**
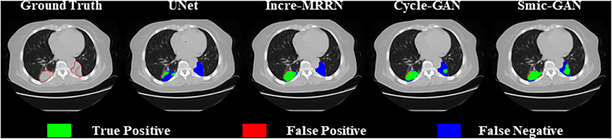
The segmentation results on one patient with symmetrical bilateral tumors.

The proposed method is trained and tested on a CT image dataset containing only single tumor patients. Clinical scenarios may occasionally present bilateral tumor cases. A challenging case where two tumors symmetrically distributed in both lungs is used to further test the robustness of the network. As illustrated in Figure 7, smic‐GAN successfully identified and segmented both tumors, achieving the highest Dice score (73.0%), while Cycle‐GAN performed worse (67.8%) and supervised networks (UNet and Incre‐MRRN) failed to detect the right‐lung tumor. Although a single instance cannot prove all possible scenarios of bilateral tumors, this particular case with symmetrical bilateral tumors does represent a most difficult situation and demonstrates the ability of smic‐GAN in handling such rare situations. Compared to Cycle‐GAN, smic‐GAN benefits from its symmetrical similarity loss, which generates more realistic and anatomically accurate healthy lung images, leading to more precise tumor segmentation. Unlike supervised networks that directly detect tumors and rely heavily on diverse training samples, smic‐GAN indirectly identifies tumors by subtracting synthetic healthy lungs, making it more robust to rare tumor locations that are underrepresented in training data.

The proposed method has several limitations. First, our method relies on 2D CT slices for network training, meaning it has not fully leveraged the spatial information present in 3D CT data. Additionally, it does not account for variations in in‐plane image resolution, which may impact feature consistency across datasets. Future research could explore the incorporation of 3D image generation to enhance performance. When extending to 3D training, differences in slice thickness must also be explicitly addressed during data preprocessing to maintain spatial consistency. Second, tuning the network parameters for smic‐GAN is more complex compared to supervised methods, as it requires balancing five different loss components. In the future, we can develop a more efficient and user‐friendly strategy for network training, which would help simplify this process. In addition, in the post‐processing stage, a 2 mm disk is applied to the image erosion process, so any tumor with a maximum diameter smaller than 4 mm would be excluded, hence limiting the application on early‐stage non‐small cell lung cancer tumor detection. In the future, we will explore the potential of directly applying neural network to improving the post‐image processing performance.

## 5 CONCLUSION

Compared to existing supervised methods, the proposed unsupervised method smic‐GAN has a similar performance but does not require manual labeling of training data. It reduces the workload of training data preparation, and also can provide a good start for manual annotation in the training of supervised networks. It may better handle rare tumor locations that are not easily collected in the training dataset.

## AUTHOR CONTRIBUTIONS

**Chengyijue Fang**: Conceptualization; methodology; formal analysis; original draft; editing. **Xiaoyang Li**: Resources; clinical assistance. **Yidong Yang**: Funding acquisition; resources; supervision; review & editing.

## CONFLICT OF INTEREST STATEMENT

The authors declare no conflicts of interest.

## Data Availability

The data in USTCFAH supporting this study are not publicly available due to patient privacy. Requests for data access may be directed to the corresponding author. The data in NSCLC‐Radiomics are publicly available (DOI: 10.7937/K9/TCIA.2015.PF0M9REI).
